# A Decade of *GigaScience*: Milestones in Open Science

**DOI:** 10.1093/gigascience/giac067

**Published:** 2022-07-12

**Authors:** Scott C Edmunds, Hans Zauner, Nicole A Nogoy, Hongling Zhou, Hongfang Zhang, Laurie Goodman

**Affiliations:** GigaScience Press, BGI Hong Kong Tech Co Ltd., 26F Kings Wing Plaza 2, 1 On Kwan Street, Shek Mun, Sha Tin, N.T., Hong Kong SAR; GigaScience Press, BGI Hong Kong Tech Co Ltd., 26F Kings Wing Plaza 2, 1 On Kwan Street, Shek Mun, Sha Tin, N.T., Hong Kong SAR; GigaScience Press, BGI Hong Kong Tech Co Ltd., 26F Kings Wing Plaza 2, 1 On Kwan Street, Shek Mun, Sha Tin, N.T., Hong Kong SAR; GigaScience Press, BGI Shenzhen, Bei Shan Industrial Zone, Yantian, Shenzhen, 518120, China; GigaScience Press, BGI Shenzhen, Bei Shan Industrial Zone, Yantian, Shenzhen, 518120, China; GigaScience Press, BGI Hong Kong Tech Co Ltd., 26F Kings Wing Plaza 2, 1 On Kwan Street, Shek Mun, Sha Tin, N.T., Hong Kong SAR

## Abstract

Open Science has gained momentum over the past decade, and embracing that, *GigaScience*, from its launch a decade ago has aimed at pushing scientific publishing beyond just making articles open access toward making the entire research process open and available as an embedded part of the publishing process. Before the journal's launch in July 2012, the editors aimed to make publishing more than a narrative presentation of work already done into a fully open process. Major milestones include creating our own data repository, embracing FAIR principles, promoting and integrating preprints, and working with other platforms to contribute to a 21st century publishing infrastructure. Almost 10 years after *GigaScience*’s launch, UNESCO published its Open Science Recommendations. With these in mind, looking back, we are happy to have contributed in various ways to UNESCO's aim to “foster a culture of Open Science and aligning incentives for Open Science” from the very beginning, and, more, to use those recommendations to guide our path into the future: to truly embrace the full spectrum of information, tools, and access to Open Science for all participants in scientific endeavours.

## Body Text

The year 2012 was labelled by some an “academic spring” as it was a major turning point where organisations began to shake up the centuries old, stale and untransparent system of scientific discourse [1]. With boycotts of closed-access publishers gathering momentum, influential policy papers signalling government moves towards open access, and the launch of a new generation of journals that were moving beyond that first important step of making published research open access to begin embracing the wider principles of Open Science. These included *F1000 Research, eLife*, and *PeerJ*, and, on the 12^th^ July 2012, *GigaScience*, which launched at the ISMB (Intelligent Systems for Molecular Biology) meeting in Long Beach [2]. While sharing many novel features, such as throwing more transparency on the black box of peer review [3], *GigaScience* also focussed on increasing transparency and reproducibility of the “Research Object” by supporting the full-spectrum of scientific research, disseminating research software and datasets by specifically crediting them as article types.

### Milestone: Our own repository for citable data and software

While “Data Journals” are not a new concept, the *Earth System Science Data* journal leveraged DataCite providing DOIs (Digital Object Identifiers) for data in 2009; with *GigaScience* most likely being the first to launch in the field of biological and biomedical science. It has been great to witness this embrace of data publication become mainstream, with Elsevier, Springer Nature, Wiley and many of the other big publishers following in our footsteps over the next few years. Where we differed from these other data publishers was leveraging the resources and expertise of our parental entity BGI (a genomics organisation) to host our own data repository, GigaDB—filling the gaps in data hosting, and providing on-hand curation and support from our in-house team of data experts (see the 10-year overview of GigaDB published alongside this piece [4]).

### Milestone: Open Knowledge and Publishing FAIR

The need to go beyond making data available was increasingly recognised, and in 2016 our Editor in Chief was one of the authors listed on the publication of the FAIR principles; a handy mnemonic recognising that data needs to be: Findable, Accessible, Interoperable, and Reusable (FAIR) [5]. The FAIR principles changed the agenda, and were embraced by the research community, policy makers and even the G20. While we hoped our research outputs were FAIR, the FAIR principles inspired us to work even harder to steward and enrich the data and metadata we were disseminating. With Plan-S stating that from 2021 all scholarly publications on the results from research funded by cOAlition S consortium (https://www.coalition-s.org/) must be published Open Access (OA), pre-empting this OA-future from our launch it has been great to see these efforts move to become the norm.

Looking back we've been pleased to see the journal receiving awards (e.g., the Association of American Publishers (AAP) 2018 Prose Awards Winner for “Innovation in Journal Publishing”) and other forms of recognition for our past efforts. But we have been very aware that our achievements in open science have been frustratingly hindered by legacy publishing infrastructure that is no longer fit for purpose in this non-print, online, and more data-centric digital age. Particularly, regarding barriers of price, speed and interactivity, this led us in 2020 to launch *GigaScience's* smaller and more agile sister journal, *GigaByte*, designed specifically for rapidly publishing work in swiftly evolving research that is well-served by available embedded content [6].

The launch of this “out-of-the-box” open research platform has hopefully been particularly timely with the biggest breakthrough signalling that Open Science has finally gone mainstream—the ratification of the United Nations Educational, Scientific and Cultural Organization (UNESCO) Open Science Recommendations in November 2021 [7]. This specifically addresses UNESCO's aim for “fostering a culture of open science and aligning incentives for open science.”

For the four key pillars highlighted by UNESCO we have particularly focussed on “Open Scientific Knowledge”: open access to scientific publications, research data, metadata, software, source code and hardware that are available in the public domain or under open licences. We've been very strict to mandate CC-BY 4.0 licences for all our textual content, CC0 public domain waivers for all our research data, OSI (open source initiative) licences for software and OSHW (open source hardware) licences for open hardware to meet this specific aim. Addressing UNESCO's call to adopt policies that require and reward open access to scientific knowledge, including scientific publications, open research data, open software, source code and open hardware.

### Milestone: Embracing preprints

To further engage with the first pillar of open access to publications, and exploit the benefits of open licences, we have gone upstream of the publication process to preprints. Encouraging use of these since our launch, and encouraging their citation; stating that we will not consider them in the evaluation of the conceptual advance of a manuscript submitted to us. We started proactively emailing bioRxiv submitters since that platform opened up preprints in the biological sciences in late-2013. Also allowing integrated B2J (bioRxiv to journal) submission, these efforts have led to over 30% of our papers having an associated preprint. As there is a logical synergism in the way preprints and open peer review speeds up and throws light on scientific communication, we experimented and were early adopters of a number of platforms, such as Publons, AcademicKarma and preprint.space. And in the last year, through connecting to the bioRxiv/medRxiv TRiP (transparent review in preprints) workflow and Sciety groups, better link and make these efforts more discoverable.

### Milestone: A 21st century publishing infrastructure

Of the other key pillars, “Open Science Infrastructures” has been an area we have also worked hard to keep on top of. UNESCO referring to shared research infrastructures (virtual or physical) that are needed to support open science and serve the needs of different communities. By making and building upon open research outputs (open text, data and code) we leveraged a whole host of third-party integrations to develop an Open Science toolkit and stack. Moving from experimenting with our GigaGalaxy.net server, Virtual Machines, and Docker containers, since 2017 we've been using the Code Ocean platform to scrutinise code, test it—allowing anyone to run it on their own cloud computing account. In addition, Protocols.io integration has allowed sharing of wet-lab protocols, and Sketchfab integration has allowed embedding and viewing (even in a virtual reality headset) of 3D models in papers.

A move to the more modern XML-only workflow in our new journal, *GigaByte*, has enabled this integration process to be even easier and quicker, aiding collaboration with Stencila in Executable Research Articles. Thus, bringing our papers to life through a whole suite of embedded widgets, that includes video, interactive maps, Hi-C genomic map viewers, and NMRium NMR spectra viewers, to date. In the physical world our in-house data curators and data scientists have proactively worked on and published case studies and experiments with the open code, data and metadata to quantify and prove the utility of these open science approaches. We have attended and hosted many workshops, hackathons and FAIRificaton “Bring Your Own Data” parties, helping to promote Open Science literacy and community-building.

### Milestone: Community engagement

The key pillar of “Open Engagement of Societal Actors” refers to extended collaboration between scientists and societal actors beyond the scientific community, and from our first year we've had a soft spot and promoted citizen and community projects, such as the “people's parrot”—a crowdfunded Puerto Rican parrot genome project [8]. In 2015 we took this a step further and launched our own community genome project, “Bauhinia Genome” (bauhiniagenome.hk) to build a bridge between science and community, and increase genomic literacy in our Hong Kong home based on crowdfunding and community education on Hong Kong's mysterious emblematic flower. This involved outreach by our team to local schools, hackerspaces, TEDx talks and even taking plant genetics onto TV and radio. Other efforts towards wider engagement have been our youtube channel and author video abstracts (https://www.youtube.com/gigasciencejournal).

The final pillar of “Open Dialogue with other Knowledge Systems” has probably been the most challenging for us to address, and while we try to meet the aims of equity and fairness and diversity and inclusiveness this is the final barrier of openness that we feel we need to work on.

### Milestone: Affordable Article Processing Charges

While OA publishers have tried to aid with costs to authors by providing discounts and waivers under specific circumstances, with some journals having extremely high APCs (article processing charges) and authors even in wealthy countries not having funding to support such costs. Seeing this is doubly troublesome when we also see the large profit margins of some publishers. At *GigaScience* and in concert with our not-for-profit publisher, Oxford University Press (OUP), we look more to covering costs with APC's than pricing APCs on what we see that the market can bear.

Still, these costs are still a barrier, and we continue to work toward lowering these with *GigaScience*. One of the main drivers of launching our sister journal, *GigaByte* was to reduce these remaining barriers. Our built-for-purpose publishing platform requires little human intervention at the publication stage, allowing us to reduce publishing costs substantially to lower our APCs well below the industry average of $2000 USD. This type of testing on speed, costs and feasibility still remains work in progress, but through doing this we can begin to look at how quickly it will be possible to open scientific communication as broadly as possible.

### Milestone: The Language Barrier

Beyond cost, though, the barrier of language remains. With most international scientific communication carried out in English, which for the majority of the world and a large proportion of scholars is not their primary language. One of the many areas of action highlighted by UNESCO is encouraging multilingualism in the practice of science, and by allowing multiple views our *GigaByte* platform has showcased dual versions of publications in Chinese [6], Spanish and Portuguese [9]. And the ability to also switch to views for visually impaired and dyslexic readers. We've also made sure to promote and use AfricaXiv and SciELO preprints [9], and link them to relevant *GigaByte* papers published by authors in Africa and Latin America.

## Conclusion

**Figure 1:**
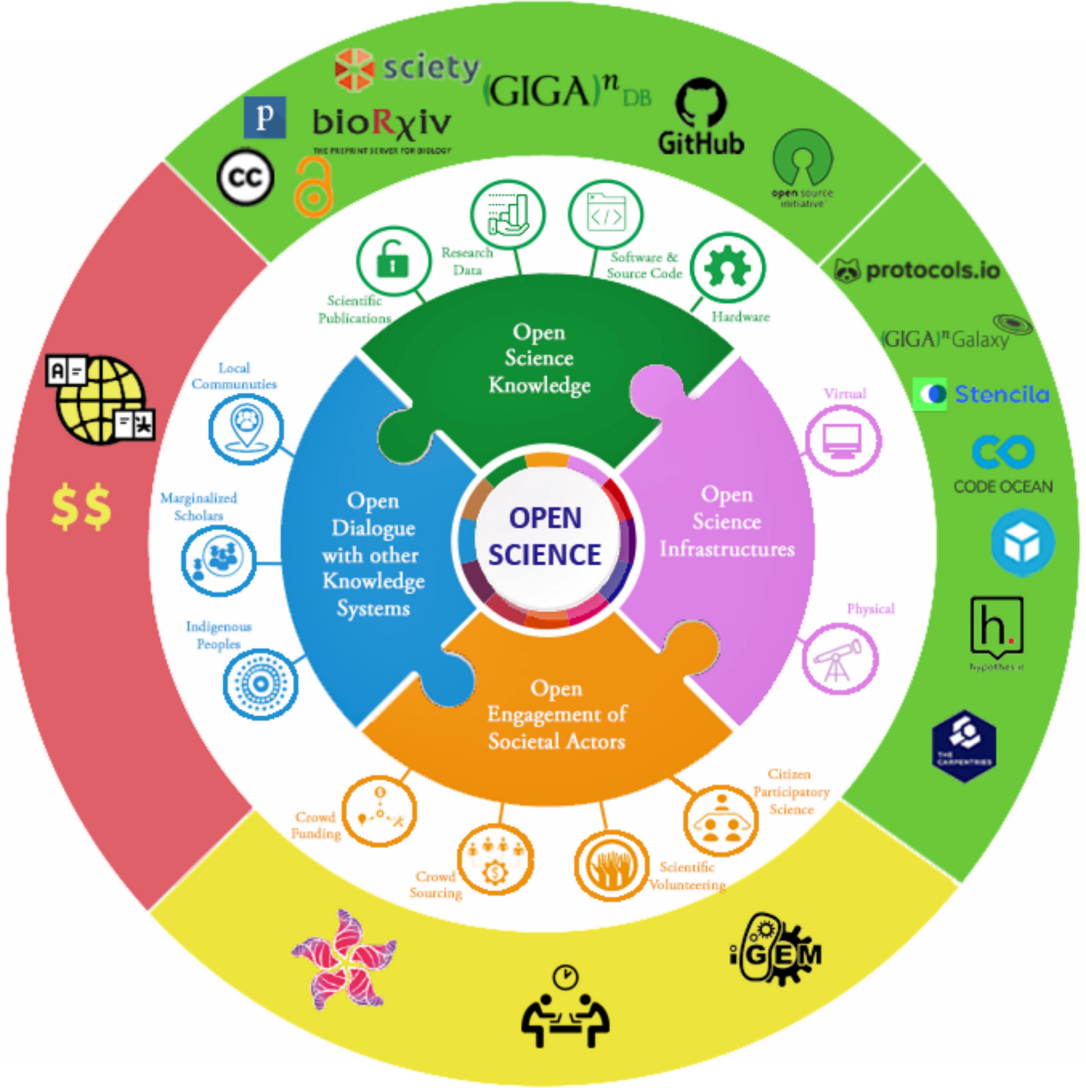
The UNESCO Open Science Recommendations lay out four pillars of Open Science (figure adapted from CC-BY graphics in the UNESCO Recommendations [7]). Since its launch 10 years ago, *GigaScience* has worked toward creating Open Science within the publishing arena. The pillars here provide a map for us to see where we've been, but also where we need to go to to continue to achieve our goals. For “Open Science Knowledge,” we have our mandated creative commons CC-BY licence for narrative content, CC0 waivers for data, and OSI and OSHW licences for open source software and hardware. We have promoted open data by developing our in-house GigaDB repository and have mandated data from our publications be hosted here or in a community supported open database. We also require authors make their source code available through GitHub. Through integration with preprint servers and open peer review platforms such as publons, bioRxiv and Sciety, we have promoted transparency and early access to knowledge. For the “Open Science Infrastructure,” we've set up third-party integrations to allow interaction with content such as protocols.io, hypothes.is, sketchfab, Stencila, CodeOcean and our GigaGalaxy.net server. Participation in the physical space has been through “Bring Your Own Data” parties and Carpentries workshops. While we have begun work on “Open Engagement of Societal Actors,” this remains a work in progress, as it has not been embedded in our publishing workflows, but we have experimented in this arena through our BauhiniaGenome citizen science projects, as well as support and promotion of hackathons and open innovation projects, such by being judges for the International Genetically Engineered Machine (iGEM) student competition. The area requiring the greatest effort for the future is under the pillar of “Open Dialogue with other Knowledge systems.” Here, we have begun that process by reducing the barriers of cost and language. While we do require authors to follow proper regulations and ethical standards via crediting indigenous and community knowledge in our processes, this is not yet actively promoted as part of our publishing activities. This fourth pillar is clearly the arena we are looking toward embracing as we move into our second decade of publishing.

After spending a decade on the front line of Open Science, the UNESCO recommendations have been a fantastic boost to our and other proponents of these practices. Signalling that Open Science has gone mainstream, it validates past efforts, and highlights what still needs to be done in the road ahead (Figure 1). Of the four key pillars our efforts have been more focussed in the areas of “Open Scientific Knowledge” and **“**Open Science Infrastructure,” and through our citizen science efforts have more recently tried to tackle “Open Engagement of Societal Actors.” The one pillar that the UNESCO recommendations have highlighted we really need to do more work on is “Open Dialogue with other Knowledge Systems.” This includes better acknowledging the rights of indigenous peoples and local communities to govern and make decisions on the custodianship, ownership and administration of data on traditional knowledge and on their lands and resources. And moving from our heavy focus on the FAIR principles, to better consider the CARE (collective benefit, authority to control, responsibility and ethics) principles [10]. Balancing the need to reduce friction in open data, with the needs of the global south to gain benefits from data built upon their traditional knowledge is a difficult task, but not an impossible one. And for our next decade it will be challenges such as these that we will hopefully be able to complete and present new milestones as these recommendations become global practice.

### Abbreviations

APC: article processing charge, CC: Creative Commons; DOI: digital object identifier, FAIR: Findable, Accessible Interoperable and Reusable; OA: Open Access; OSHW: Open Source Hardware; OSI: Open Source Initiative; UNESCO: United Nations Educational, Scientific and Cultural Organization.

### Data Availability

Not applicable.

## Funding

Not applicable.

## Editor's Note

This commentary is part of a series to celebrate a Decade of *GigaScience*, to coincide with the 10th anniversary of *GigaScience* journal's launch in July 2012. These papers take a look back at 10 years of advances in large-scale research as Open Science has become mainstream.

## Competing Interests

All of the authors work for GigaScience Press.
